# Comminuted Mason III/IV Radial Head Fractures: What Is the Best Treatment Between Prosthesis and Radial Head Resection? A Systematic Review and Meta-Analysis

**DOI:** 10.3390/jcm14051773

**Published:** 2025-03-06

**Authors:** Luca Bianco Prevot, Livio Pietro Tronconi, Vittorio Bolcato, Riccardo Accetta, Stefania Fozzato, Giuseppe Basile

**Affiliations:** 1 IRCCS Ospedale Galeazzi—S. Ambrogio, 20157 Milan, Italy; riccacc@gmail.com (R.A.); basiletraumaforense@gmail.com (G.B.); 2 Residency Program in Orthopaedics and Traumatology, University of Milan, 20122 Milan, Italy; 3 Department of Human Science, European University of Rome, 00163 Rome, Italy; liviopietro.tronconi@unier.it; 4 Maria Cecilia Hospital, GVM Care & Research, 48033 Cotignola, Italy; 5 Maria Beatrice Hospital, GVM Care & Research, 50121 Firenze, Italy; 6 Presidio Ospedaliero di Rho—ASST Rhodense, 20017 Rho, Italy; ste.fozzato@gmail.com

**Keywords:** radial head fractures, Mason III/IV, meta-analysis

## Abstract

**Background/Objectives**: Various surgical methods have been proposed for the treatment of comminuted Mason III/IV radial head fractures. In particular, the advantages and disadvantages between prosthesis implantation (RHA) or radial head resection (RHR) are not sufficiently quantified in the current literature. **Methods**: A systematic literature search was conducted using PubMed Web of Science, Cochrane Library, and Embase in February 2024. Studies conducted on patients with Mason type III or IV radial head fractures and studies relating to surgical methods, including radial head resection or Radial head prosthesis implantation, were included. The two methods were evaluated in terms of clinical and functional results through the DASH score (Disability of the arm, shoulder, and hand), Mayo Elbow Performance Index (MEPI), and flexion-extension range of motion. The onset of osteoarthritis and complications were also assessed. Risk of bias and quality of evidence were assessed using Cochrane guidelines. **Results**: A total of 345 articles were evaluated and, of these, 21 were included in the study for a total of 552 patients. The results of the meta-analysis showed no significant differences in favor of RHA or RHR in terms of Mayo Elbow Performance (*p* = 0.58), degrees of flexion (*p* = 0.689), degrees of extension deficit (*p* = 0.697), and overall incidence of complications (*p* = 0.389), while it highlighted a statistically significant difference in terms of DASH score (19.2 vs. 16.2, respectively; *p* = 0.008) and subjects who developed osteoarthritis (13.4% vs. 47.3%, respectively; *p* = 0.046). **Conclusions**: The results of this meta-analysis confirm that both surgical methods provide good functional outcomes, with no significant differences in MEPI, DASH, and range of motion. However, a higher incidence of post-traumatic osteoarthritis was observed in patients undergoing RHR. Additionally, RHR patients exhibited slightly worse functional outcomes in the DASH score; however, this difference is not substantial enough to be considered clinically significant. These findings suggest that while both techniques are viable, RHA may be preferable in patients at higher risk of joint degeneration and instability, and the choice of treatment should be tailored to individual patient characteristics.

## 1. Introduction

Fractures of the radial head are the most common type of fractures affecting the elbow joint, having an incidence of approximately 2.8 cases per 10,000 people per year [1].

The classification that is most frequently used in describing this type of fracture is the one proposed by Mason in 1954, in which 4 grades are identified depending on the type of fracture, the fragments, and the decomposition [2].

Comminuted Mason type III and type IV fractures of the radial head require surgical treatment and, even if correctly treated, can lead to various complications, such as joint stiffness, joint instability, formation of heterotopic ossifications, nervous compressive phenomena, non-union of the fracture, and secondary decompositions, events that can limit the quality of life of patients in terms of lower satisfaction and well-being in relation to the different spheres of daily life and in relation to relational and work aspects, as well as increasing the risk of further surgical interventions [3].

These fractures are commonly associated with other elbow injuries, such as fractures of the capitellum and coronoid and/or rupture of the ligaments, both medial and lateral, and of the interosseous membrane [4]. The primary objective of surgical treatment is to restore elbow stability to preserve the complex physiological kinematics of the elbow.

There are various surgical treatments proposed for these types of fractures; fixation with plate and screws can be a surgical solution in those fractures in which an adequate reduction of the fragments can be achieved, especially in the young population [5]; however, there is a risk of secondary breakdown, intolerance to the means of fixation, and of non-union of the fracture.

When fixation is not feasible, two alternative options exist: Radial Head Arthroplasty (RHA) and Radial Head Resection (RHR).

RHA allows maintaining good kinematics of the joint and good stability of the elbow; various studies demonstrate how the prosthesis has comparable, if not superior, results in terms of outcomes compared to synthesis with plate and screws [6].

RHR can be a treatment choice in these patients as it is a rapid intervention with good clinical results, especially in the elderly population. RHA and RHR do not appear to be complication-free surgical procedures; the first presents the risk of malpositioning, mobilization, overstuffing, and/or disassembly of the prosthesis, complications that can lead to implant failure with the need for subsequent revision surgery [7]; while the second has a risk of around 35–40% of developing arthrosis of the elbow joint and instability [8].

Therefore, regarding the most appropriate surgical treatment of Mason type III and IV fractures of the radial head with high comminution, both methods are widely used in clinical practice with controversial findings, and the debate on the most suitable approach remains open.

The aim of this work was to evaluate and quantify what is the best treatment to offer to the patient with Mason type III and IV radial head fracture, between the implantation of the prosthesis and the resection of the radial head, in terms of clinical outcomes and onset of complications.

## 2. Materials and Methods

This systematic review was drafted using the Preferred Reporting Items for Systematic Reviews and Meta-analysis (PRISMA) guidelines [9].

This systematic review has been registered and listed in the PROSPERO database (CRD42024511233) under the National Institute for Health Research, University of York, Centre for Reviews and Dissemination. A comprehensive literature search was conducted on 2 May 2024, utilizing PubMed, Web of Science, and Embase with the following search string: (radial head fracture) AND (mason type III OR mason type 3 OR mason type 4 OR mason type IV) AND (arthroplasty OR prosthesis OR replacement) AND (resection OR excision).

Duplicates were removed and all records were subsequently assessed for suitability based on the abstract title and, if necessary, the full text. The inclusion criteria that were chosen were: studies conducted on patients with Mason III or IV radial head fractures, studies relating to surgical methods including radial head resection or implantation of radial head prosthesis (RHA); level of evidence I, II or III; human studies; written in English. No time limit was considered in the inclusion criteria.

Exclusion criteria were: studies conducted on subjects with Mason I or II radial head fractures, preclinical studies or ex vivo studies, level of evidence IV; meta-analysis; systematic literature reviews; articles written in other languages.

Article selection was carried out independently by two authors (L.B.P., S.F.), following the inclusion criteria. Any disagreements were addressed through discussion with a third author (G.B.), who facilitated resolution by reviewing the issue comprehensively.

### 2.1. Data Extraction

Data extraction was independently carried out by two authors (L.B.P., S.F.) using the full-text articles or Supplementary Materials. Collected information included study methodology, such as study type, level of evidence, year of publication, and fracture classification based on the Mason system, the type of operation the patients had undergone, the type of prosthesis used, and the duration of follow-up.

Patient characteristics and clinical outcomes of treatments were also collected: number of patients included and evaluated at follow-up, sex, age, post-surgical clinical scores (Mayo Elbow Performance Index (MEPI), The Disabilities of the Arm, Shoulder and Hand (DASH), Broberg and Morrey Score), reported complications, appearance of osteoarthritis during follow up according to the Broberg and Morrey classification [10], degrees of flexion, pronation, and supination, extension deficit expressed in degrees, and number of subjects who developed osteoarthritis (OA) in follow-up. The functional outcomes collected were included if acquired after at least one year of follow up as it was considered that after this period of time, the post-operative results achieved by the patients could be considered stabilised.

Since some data were missing or could not be extrapolated due to the heterogeneity of the clinical studies and the population sample analyzed in the various studies, data were considered missing in the presentation of our results.

### 2.2. Quality and Risk of Bias Evaluation

Two independent authors (L.B.P. and S.F.) conducted the risk of bias and quality assessment, with any discrepancies resolved through discussion and consensus involving a third author (R.A.). The studies included in the meta-analysis were assessed using the revised Risk of Bias tool for randomized trials (RoB 2.0) and the Risk of Bias in Non-Randomized Studies of Interventions (ROBINS) tool for non-randomized clinical trials, following Cochrane’s recommendations [11].

The overall quality of evidence for each outcome was graded as high, moderate, low, or very low according to GRADE (Grading of Recommendations Assessment, Development and Evaluation) guidelines [12].

### 2.3. Statistic Analysis

Meta-analyses were performed only with retrospective comparative studies and randomize control trials to evaluate which treatment is more effective between RHA and RHE. Differences between the management of comminuted Mason III and IV radial head fractures by RHA or RHR in terms of MEPD and DASH, flexion, and extension deficits were evaluated by the inverse variance method and expressed as mean differences for continuous variables (MD = RHA-RHR mean difference), while the onset of osteoarthritis and the appearance of post-operative complications were evaluated through the Mantel–Hanszel test and expressed as risk ratios for dichotomous variables (RR = RHA/RHR risk ratio). Statistical analysis was performed with the PythonMeta (version 1.26) package in Python (version 3.9).

The I^2^ statistic was utilized to evaluate heterogeneity, considering values above 25% as significant. Following the approach suggested by Borenstein et al. [13], a random-effects model was applied for the meta-analysis, assuming that substantial differences across studies did not support the use of a fixed-effects model. If I^2^ was below 25%, the meta-analysis was re-conducted using a fixed-effects model. A significance threshold of 0.05 was established for the two-sided test analysis. When standard deviations were not provided in the full-text articles, they were estimated using the sample interval, as recommended by Walter and Yao [14].

### 2.4. Selection of Articles

**Figure 1:**
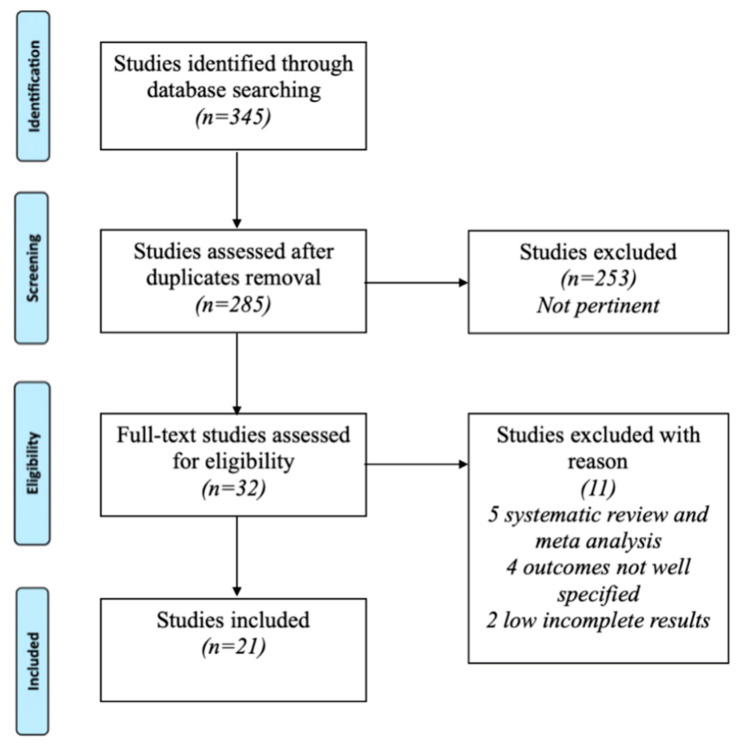
Shows the illustrating flowchart of the selection process of the articles.

The PRISMA flowchart illustrating the article selection process is presented in Figure 1. The literature search yielded 259 articles from PubMed, 26 from Embase, and 60 from Web of Science. After removing 50 duplicate records, 253 articles were excluded based on title and abstract screening. Among the 32 remaining studies, 11 were further excluded for not meeting the inclusion criteria, resulting in a final selection of 21 articles for analysis.

Among the analyzed studies, 12 were retrospective case series, 8 were retrospective comparative studies, and 1 was a randomized controlled trial. In 12 studies, patients with Mason type III fractures were included, while 8 studies considered both Mason type III and IV fractures. One study specifically focused on patients with Mason type IV fractures.

In nine studies, patients undergoing RHA were compared with those undergoing RHR. In eight studies, the results of patients operated with RHA implantation were presented. In four studies, the results of patients treated with RHR were presented.

In total, a population of 552 patients suffering from multifragmentary radial head fractures were analyzed, of which 209 underwent radial head resection surgery while the other 343 underwent surgery with radial head prosthesis implantation.

## 3. Results

### 3.1. Results Description

The patients treated with RHA implantation had an average age of 48.4 years and 164 were females and 179 males, while the patients treated with RHR had an average age of 51.3 years and 111 were females and 98 males.

The mean follow-up was 57.2 months with a range from 12 to 312 months.

To evaluate long-term outcomes (>12 months after treatment), the MEPI score was used in all the studies examined, the VAS score in 4 studies, the DASH score in 11 studies, and the Broberg and Morrey Score in 3 studies.

Patients with RHA implantation had an average MEPI of 89.8 (range 78.9–96.4), an average DASH score of 19.2 (range 6.6–28.7), and an average Broberg and Morrey score of 85.8 (range 77.4–94.2), while patients undergoing RHR had Mean MEPI of 87 (range 74–96.6), mean DASH score of 16.2 (range 6.6–34), and mean Broberg and Morrey score of 82.6 (range 79.2–88).

Patients treated with RHA implant had an average flexion of 126.5° (range 120–145), an average extension deficit of 14.4° (range 1.3–24), an average pronation of 73.7 (range 58.5–83.7), and an average supination of 72.2 (range 58.8–85.9), while patients treated with RHR had an average flexion of 130.9° (range 104.3–150), an average extension deficit of 11.8° (range 4.4–25), an average pronation of 76.7 (range 56.4–85), and mean supination of 75.2 (range 60.6–84.6).

In cases treated with RHA, the onset of grade I sec osteoarthritis was found in 38 patients (11.1%), grade II in 6 patients (1.74%), and grade III in 2 patients (0.6%).

In cases treated with RHR, the onset of grade I sec osteoarthritis was found in 62 patients (29.7%), grade II in 35 patients (16.7%), and grade III in 2 patients (0.9%).

In total, 64.3% of the implanted capital prostheses were monopolar prostheses while 35.7% of the prostheses were bipolar.

Different complications were found in the two groups. Eighty-three patients undergoing RHA implantation (24.2%) developed complications, while thirty-nine patients undergoing RHR (18.7%) developed complications. Full RCT and case control studies details are summarized in Table 1 while case series studies details are summarized in Table 2.

### 3.2. Meta-Analysis Outcomes: MEPS, DASH, Elbow Flexion and Extension

**Figure 2:**
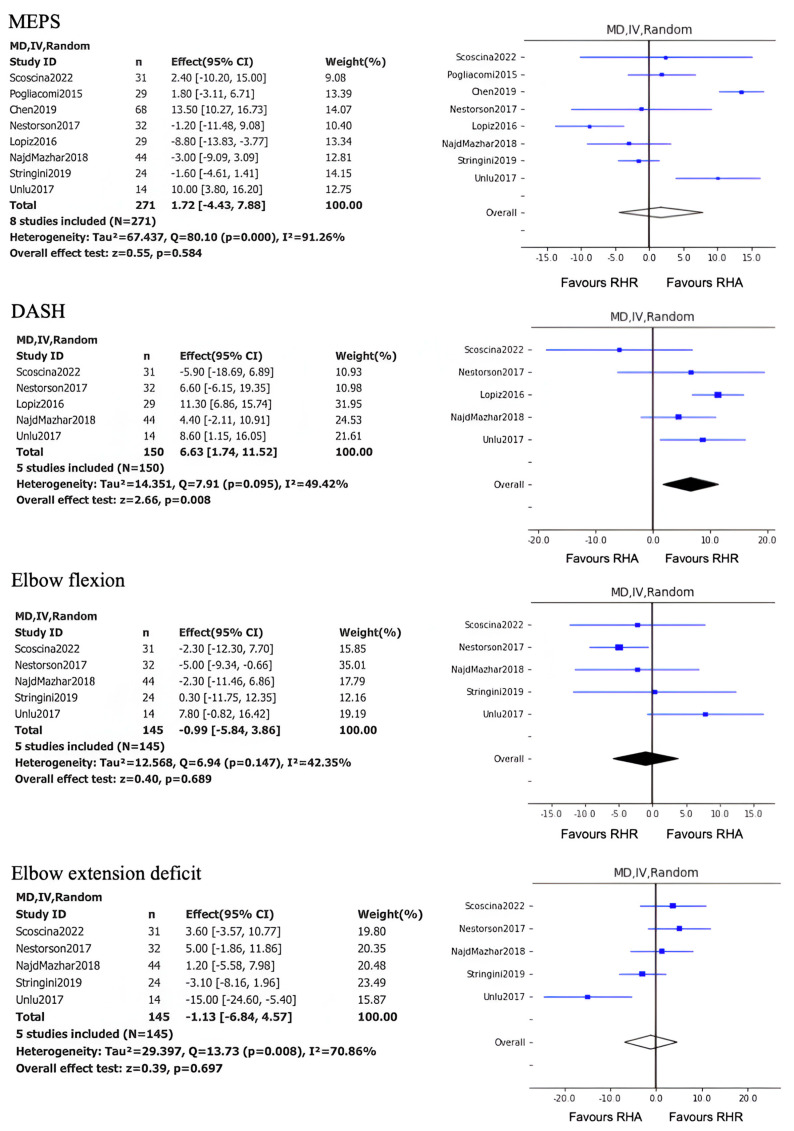
Forest plots for MEPS, DASH, and Elbow flexion and extension, CI = confidence interval, MD = mean difference.

The results of the meta-analysis conducted on nine studies (eight level 3 studies and one level 2 study) showed no differences in terms of MEPI score with MD, 1.7 (95% CI, −4.4 to 7.9; *p* =0.584). The results of the meta-analysis conducted on five level 3 studies showed significant differences in favor of radial head resection in terms of postoperative DASH score with MD, 6.6 (95% CI, 1.7 to 11.5, *p* = 0.008). The results of meta-analyses conducted on five level 3 studies showed no significant differences in terms of postoperative elbow flexion score with MD, −0.99 (95% CI, −5.84 to 3.86, *p* = 0.689), and deficit of postoperative elbow extension with MD, −1.3 (95% CI, −6.84 to 4.57; *p* = 0.697) (Figure 2).

### 3.3. Meta-Analysis Complications

The results of the meta-analysis conducted on five level 3 studies showed a statistically significant difference in favor of the implantation of radial head prosthesis in terms of the onset of elbow osteoarthritis (*p* =0.046).

**Figure 3:**
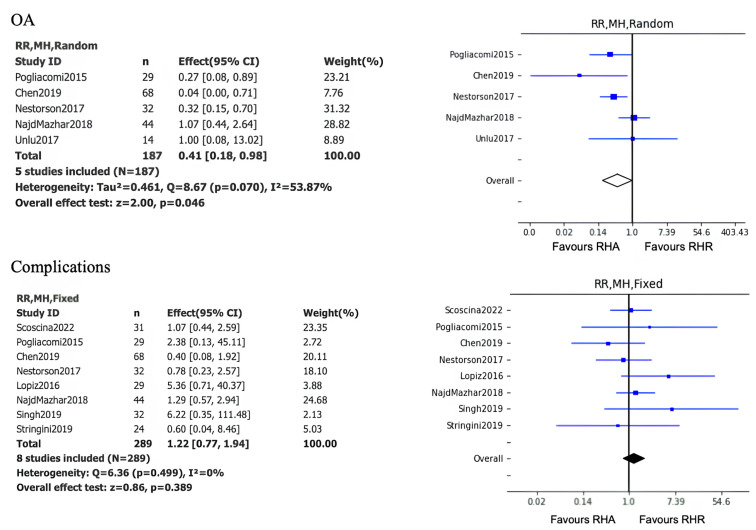
Forest plots for OA and Complications, CI = confidence interval, RR = risk ration.

Instead, the results of the meta-analysis conducted on eight level 3 studies did not show a statistically significant difference between the two methods in terms of the onset of post-operative complications (*p* =0.389) (Figure 3).

### 3.4. Risk of Bias and Quality of Evidence

The articles analyzed for this paper were 12 case series (evidence level 4), 8 comparative studies (evidence level 3), and 1 RCT study (evidence level 2).

The risk of bias was rated moderate (“some concerns”) in the randomized trial: the main source of bias was the lack of blinding of the assessor. The risk of bias of the overall analysis was rated “moderate” for all 8 non-randomized studies. The main problem was the presence of uncontrollable confounding factors: the level of activity after the operation, the type of rehabilitation, the prescription of braces in the post-operative period, and the time elapsed between the fracture and the operation could be different in two groups and this could seriously influence the results at follow-up.

The level of evidence was considered low for the MEPI, for the DASH, for the degrees of flexion and extension deficit, and for the risk of onset of osteoarthritis.

**Figure 4:**
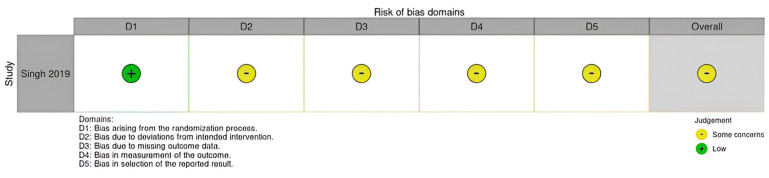
Risk of bias assessments according to RoB 2.0 tools.

**Figure 5:**
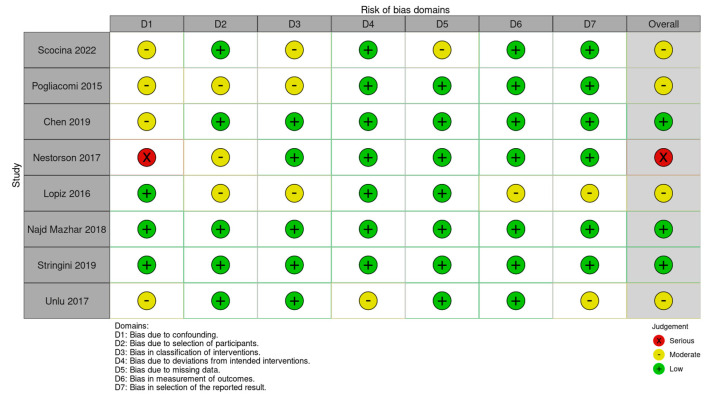
Risk of bias assessments according to ROBINS tools.

Details of risk of bias assessments are listed in Figure 4 and Figure 5.

## 4. Discussion

This systematic literature review and meta-analysis compared the results of the surgical management of comminuted Mason type III and IV fractures of the radial head between patients treated with prosthesis implantation with those treated with radial head resection, demonstrating no significant differences in terms of patient-reported outcomes, functional outcomes, and the onset of post-operative complications, but identified that the development of post-traumatic elbow arthritis is more frequent in those patients undergoing RHR.

There are several articles in the literature that explore the results of surgical treatment of comminuted Mason III radial head fractures [36,37,38]. The biggest controversy concerns the choice of the best treatment for this type of fracture. Some authors argue that the choice with better post-operative results is open reduction and internal fixation (ORIF). However, internal fixation techniques are challenging and time-consuming in the presence of multiple fragments; furthermore, these fractures are very difficult to repair due to the poor quality of the bone and/or the inadequate fixation of very small fragments. For this reason, it is often necessary to resort to resection of the radial head or the implantation of a radial head prosthesis. There is no consensus in the literature on which is the most suitable choice to obtain better long-term functional results and a lower complication rate.

This meta-analysis offered important insights regarding which treatment option is most suitable to choose between RHA and RHR. Regarding the functionality indices of the operated limb, there was conflicting evidence. In fact, regarding the results of the Mayo Elbow Performance Index, no statistically significant difference was found in favor of one or the other surgical approach, while that relating to the DASH score showed a statistically significant difference in favor of the approach with resection of the radial head, with an average of 6.6 points less in favor of the latter technique. This difference could be attributable to the different areas explored by the two scores. The MEPI is a questionnaire that considers both subjective parameters such as pain and daily function, while at the same time taking into account objective medical parameters such as range of motion (ROM) and elbow stability. In contrast, the DASH score is based on 30 questions regarding the overall functionality of the upper limb, making it less specific for elbow pathologies [39]. Moreover, the difference in DASH scores between the two groups is smaller than the minimal clinically important difference [40]. Therefore, this result must be interpreted critically, stating that there is no certain superiority between RHA and RHR in terms of functionality but that both methods produce good results in terms of functional outcomes.

Another interesting aspect that emerged from this meta-analysis is the data relating to the ROM of the operated elbow; neither the data relating to elbow flexion nor those relating to the extension deficit demonstrated a statistically significant difference between the two surgical methods. Both surgical methods reported average degrees of post-operative flexion, which were around the minimum values of the normal flexion range of a healthy elbow (which is approximately 130–154°), and average degrees of extension deficit, which was around the upper values of the normal range of extension of a healthy elbow (which are approximately −6–11°). These data support even more the fact that, from a functional point of view, RHA and RHR have overlapping results.

Another important aspect to consider is the reduction in strength that may be encountered following surgical treatment of these fractures.

Patients should assess whether they can manage a reduction in their daily activity level. In the study by Unlu et al. [23], grip strength was compared between patients undergoing RHA and RHR. The findings indicated that those treated with RHA retained an average grip strength of 77.8% relative to the contralateral limb, whereas patients who underwent RHR exhibited an average grip strength of 47.8% in comparison to the unaffected limb. As a result, radial head resection should primarily be considered for older patients with lower functional demands or for individuals who do not require full grip strength for their daily tasks.

Beyond functional ability and the capacity to adapt to a noticeable strength deficit, another key factor in determining the most appropriate treatment option is the potential risk of adverse events.

This meta-analysis offered important insights into this topic. The percentage of complications found in the group of patients treated with RHA was 24.4% while that of patients treated with RHR was 18.7%. The complications most frequently encountered in patients undergoing RHR are heterotopic ossifications, elbow instability, and stiffness, while in subjects undergoing RHA implantation, the most frequent complications were heterotopic ossifications, rigidity, and neuropathy of the ulnar nerve. It is interesting to note how two of the articles analyzed, that of Nosenzo et al. [29] and that of Marsh et al. [20], highlighted 35 cases of periprosthetic lucency in 72 operated patients (48.6%).

This finding, however, does not correlate with poor functional outcomes or an increased rate of implant revision.

The meta-analysis did not highlight any statistically significant difference between the two methods in terms of the onset of post-operative complications.

The analysis instead revealed a statistically significant difference in favor of RHA implantation in terms of risk of onset of post-traumatic elbow arthritis, with an overall incidence in subjects with RHA of 13.8% and an overall incidence in subjects with RHR of 47.3%. Both RHA and RHR can lead to a rapid onset of elbow arthritis for different reasons; radial head prostheses, although useful for stability, can sometimes present congruence problems with the native joint, generating anomalous distributions of forces and accelerating the degenerative process. Furthermore, precision in implant placement plays a crucial role; even minimal misalignment can lead to premature wear of the articular cartilage.

On the other side, the radial head is the secondary stabilizer for valgus stress (the ulnar collateral ligament is the primary stabilizer) and is the primary stabilizer for longitudinal stability of the joint (the interosseous membrane is the secondary stabilizer) [41].

Biomechanical studies have demonstrated how resection of the radial head can cause even subclinical elbow instability, which can lead to the development of post-traumatic arthrosis [42], so much so that this procedure is contraindicated in the context of lesions of the ulnar collateral ligament or of the membrane interossea, unless these lesions are treated simultaneously.

It is therefore important to emphasize that, especially in Mason type IV fractures, where an elbow dislocation occurs and consequently a ligament injury, ensuring adequate joint stability is essential in cases where radial head resection is performed. This can be achieved by considering, if necessary, an additional surgical procedure for ligament repair [18,43,44].

Both surgical methods represent valid alternatives when dealing with patients with comminuted fractures of the radial head. RHR is a safe, fast technique, with a rapid learning curve, which allows us to obtain good functional results and a low incidence of complications, but which nevertheless exposes patients to a greater risk than RHA implantation of developing elbow arthrosis and loss of strength of the involved limb.

The radial head prosthesis also allows for good functional results and good elbow stability but has a higher incidence rate of post-operative complications than RHR.

The available literature had numerous limitations that were inevitably reflected in this meta-analysis. We did not only identify published RCTs comparing these two surgical techniques; most of the literature involved low-quality studies with a high risk of bias and low to very low evidence. Future research efforts should focus on randomized trials comparing different operative approaches to provide higher-quality evidence and minimize the risk of bias. Additionally, further studies should investigate long-term follow-ups and implant longevity to enhance understanding of patient outcomes over time. Furthermore, in the various studies analyzed, heterogeneous post-operative rehabilitation protocols and post-operative indications were used. An important limitation of our study is represented by the heterogeneous nature of the patient populations considered. While some studies have included patients with isolated radial head fractures, others have evaluated patients with fractures associated with a ‘terrible triad’, which requires a different surgical approach. This heterogeneity could influence the comparability of results between different types of treatment.

The included patient cohort was homogeneous in terms of age, gender, and treatment, which reinforces the validity of the findings and enables this meta-analysis to offer a thorough and reliable perspective on the subject. While further high-quality studies are warranted, the results of this meta-analysis provide a clear assessment of the strengths and limitations of the existing literature, identify key areas for future investigation, and offer valuable guidance for both surgeons and patients in selecting the most appropriate treatment for Mason III and IV radial head fractures. Additionally, these findings support the implementation of preventive strategies to minimize complications—not only to enhance clinical outcomes and patient quality of life but also to mitigate the risk of potential medico-legal issues. It is essential to provide the patient with a detailed explanation of the available therapeutic options, with their associated risks and benefits, exposing in a manner, that is both clear and understandable, every useful element in order to obtain valid and informed consent [45].

## 5. Conclusions

This systematic literature review and meta-analysis compared the results of surgical management of comminuted Mason type III and IV fractures of the radial head between patients treated with prosthesis implantation with those treated with radial head resection and demonstrated how both the methods produce adequate functional results in patients. It has also emerged that there is a difference in terms of risk of developing post-traumatic elbow arthritis. The results of our study show that surgical options for radial head fractures are varied and depend on the specific clinical context of the patient. Therefore, surgical decisions must be individualized based on the patient’s presentation, taking into account the presence or absence of instability.

The management of comminuted radial head fractures represents a challenge for orthopedic surgeons, as it requires careful evaluation of the therapeutic options available in relation functional results and possible post-operative complications.

## Figures and Tables

**Table 1:** Characteristics of RCT and case-control studies included in this review. (RHA = radial head arthroplasty; RHR = radial head resection; SD = standard deviations; OA = osteoarthritis; MEPI = Mayo Elbow Performance Index; DASH = The Disabilities of the Arm, Shoulder, Hand; n.a = not applicable. We report, when available, standard deviations or range, express between the brackets).

Authors	Mason Classification	Total Patients	Mean Age	Treatment	Complications	MEPI	DASH	Flexion	Extention
Scoscina et al. 2022 [15]	III	31	53.9 SD 7.6	15 RHA	2 instability 2 heterotopic ossification 2 osteolysis	85.7 SD 17.5	28.7 SD 16.8	124.1 SD 16.4	20.7 SD 11.1
			64.5 SD 6.8	16 RHR	3 instability 3 heterotopic ossification	83.3 SD 18.3	34.6 SD 19.5	126.4 SD 11.4	17.1 SD 9.1
Pogliacomi et al. 2015 [16]	III/IV	29	58.4 (51–63)	20 RHA	2 superficial wound infection3 OA	90.5 SD 5.1	n.a	n.a	n.a
			66.7 (65–74)	9 RHR	0	88.7 SD 6.7	n.a	n.a	n.a
Chen et al. 2019 [17]	III	68	37.47 SD 6.7	34 RHA	1 heterotopic ossification 1 stifness	94.24 SD 3.4	n.a	n.a	n.a
			35.94 SD 5.7	34 RHR	5 heterotopic ossification	80.65 SD 9	n.a	n.a	n.a
Nestorson et al. 2017 [18]	IV	32	56 (19–79)	18 RHA	1 instability3 aseptic loosening of the prostetic 1 complex regional pain syndrom5 OA	86.95 SD 10.7	23.88 SD 20.9	145 (125–155)	20 (0–30)
			50 (29–70)	14 RHR	2 stiffnes2 instability12 OA	88.21 SD 17.2	17.25 SD 15.9	150 (135–155)	15 (0–45)
Lopiz e al. 2016 [19]	III	25	54.4	14 RHA	3 stifness2 neurapraxia (1 ulnar e 1 radial nerv)	78.9	24.8 (0–27)	n.a	n.a
			53.7	15 RHR	1 stifness	87.7	13.5 (0–21)	n.a	n.a
Mazhar et al. 2018 [20]	III/IV	44	36 SD 9.4	15 RHA	1 ullnar nerv neuropathy1 radio-ulnar synostosis4 heterotopic ossification5 OA	88.7 SD 9.9	13.9 SD 11.8	131.3 SD 13	19 SD9
			40.7 SD 13.6	29 RHR	1 radio-ulnar synostosis8 heterotopic ossification8 OA	91.7 SD 9.5	9.5 SD 7.1	133.6 SD 17.5	17.8 SD 13.8
Singh et al. 2019 [21]	III	32	42.9 SD 14.4	17 RHA	2 stifness1 instability	90.7 SD 8.0	n.a	n.a	n.a
			43.9 SD 17.1	15 RHR	0	85.8 SD 10.7	n.a	n.a	n.a
Strigini et al. 2019 [22]	III	24	48 (30–72)	15 RHA	1 instability	95 (80–100)	n.a	120.3 (70–140)	1.3 (0–10)
			57.3 (35–81)	9 RHR	2 instability	96.6 (90–100)	n.a	120 (90–140)	4.4 (0–30)
Unlu et al. 2017 [23]	III	14	42.5	7 RHA	1 OA	84	25.8	112.1 SD 10.3	10 SD 8.7
			49	7 RHR	1 OA	74	17.2	104.3 SD 5.4	25 SD 9.6

**Table 2:** Characteristics of the case series studies included in this review. (RHA = radial head arthroplasty; RHR = radial head resection; SD = standard deviations; OA = osteoarthritis; MEPI = Mayo Elbow Performance Index; DASH = The Disabilities of the Arm, Shoulder, Hand; n.a = not applicable. We report, when available, standard deviations or range, express between the brackets).

Authors	Mason Classification	Total Patients	Mean Age	Treatment	Complications	MEPI	DASH	Flexion	Extention
Tarallo et al. 2017 [24]	III	31	49 (31–80)	31 RHA	8 heterotopic ossification	91.384 SD 10.2	n.a	125.2 SD 14	13.2 SD 13.1
Van Hoecke et al. 2016 [25]	III/IV	21	53.2	21 RHA	1 perirposthetic lucency	88.6 (50–100)	23.1 (0–63)	121.8 (110–140)	24.8 (15–40)
Marsh et al. 2016 [26]	III	55	53 SD 14	55 RHA	20 heterotopic ossification 25 perirposthetic lucency 21 OA	91 SD 13	14 (0–55)	134 SD 9	13 SD 14
Ricon et al. 2012 [27]	III	28	54 SD 5.5	28 RHA	2 overstuffig 5 heterotopic ossification 11 radial head resorpion 1 ullnar nerv neuropathy	92 (70–100)	n.a	120 (100–135)	15 (0–55)
Kandel et al. 2022 [28]	III/IV	22	38 SD 10.9	22 RHA	3 superficial wound infection 1 stifness	96.4 SD 7.7	n.a	115.9 SD 9.2	n.a
Nosenzo et al. 2020 [29]	III	17	56 (31–74)	17 RHA	10 heterotopic ossification 9 perirposthetic lucency	91.2 (70–100)	6.6 (0–34)	132 (105–140)	17 (0–60)
Mou et al. 2015 [30]	III	12	40.9 (20–70)	12 RHA	2 heterotopic ossification 1 OA	97.1 (94–100)	11.9 (0–25)	130 (110–140)	n.a
Baek et al. 2020 [31]	III/IV	24	49.8 (19–73)	24 RHA	4 heterotopic ossification 6 perirposthetic lucency 7 OA	88.7 SD 11.5	19.4 SD 7.8	132.7 SD 7.4	4.7 SD 10.8
Iftimie et al. 2011 [32]	III/IV	22	37 (18–61)	22 RHR	8 heterotopic ossification 22 OA	94 (85–100)	n.a	135 (120–145)	5 (0–30)
Antuna et al. 2010 [33]	III/IV	26	29 (15–39)	26 RHR	26 OA	95 (60–100)	n.a	139 (100–145)	9 (0–30)
Lindenhovius et al. 2007 [34]	III/IV	15	48 (25–69)	15 RHR	3 elbow dislocation 8 OA	86 (35–100)	15 (0-58)	136 (115–150)	6 (0–30)
Yalcinkaya et al. 2013 [35]	III	14	38.8 (20–67)	14 RHR	3 heterotopic ossification 7 OA	88.6 (75–100)	6.6 (0-15)	133.9 (120–145)	6.9 (0–20)

## Data Availability

Not applicable.
